# COVID-19 Information Sources and Vaccination Status Among Californian Adults by Generation Using the 2022 California Health Interview Survey: Cross-Sectional Study

**DOI:** 10.2196/85904

**Published:** 2026-02-17

**Authors:** Julia Forest Zabala, Melissa Sablik, Gina R Finical, Victoria F Keeton, Janice F Bell

**Affiliations:** 1 Betty Irene Moore School of Nursing, University of California, Davis, 2570 48th Street, Sacramento, CA, 95817, United States, 1 (916) 734-2145

**Keywords:** COVID-19 vaccines, COVID-19, self-report, pandemics, health literacy, social media, information sources, vaccination hesitancy, generational membership, media

## Abstract

**Background:**

As communication technology advances and the digital divide grows, a deeper understanding of the influence of different information sources on vaccine uptake by generations can inform targeted public health interventions in times of future crisis. While the COVID-19 pandemic highlighted the role of media sources on the decision to receive vaccines, no studies have focused on the impact of the type and number of information sources in a population-based sample in California.

**Objective:**

In this study, we examined associations between Californians’ self-reported most relied upon COVID-19 information sources, categorized by type and measured as a count, and their COVID-19 vaccination status using data collected from the 2022 California Health Interview Survey. To address differences in information preferences and vaccine uptake by age, we also tested for potential effect modification of the relationship between relied upon COVID-19 information sources and vaccination status by generational membership (eg, Generation Z, millennials, Generation X, baby boomers, and Silent Generation).

**Methods:**

We conducted a secondary analysis of cross-sectional data from the 2022 California Health Interview Survey. Vaccine status (any or none) was modeled as a function of information sources (or count) controlling for important sociodemographic and health confounding variables. Interaction terms of information sources (or count) by generational status were added to the models to test effect modification, and if significant, the models were stratified by generation. All analysis was survey-weighted to account for the complex survey sampling design.

**Results:**

Compared to relying on traditional news media for COVID-19 information, relying on word of mouth (odds ratio [OR] 0.6), social media (OR 0.62), and doctors (OR 0.41) for COVID-19 information was associated with lower odds of being vaccinated for COVID-19. A dose-response relationship was identified, with each additional information source associated with 9% higher odds of being vaccinated for COVID-19. In stratified models, social media, compared to traditional news media, was associated with lower odds of vaccination for Generation X, baby boomers, and the Silent Generation.

**Conclusions:**

Health information preferences, especially for traditional news media, are associated with COVID-19 vaccine uptake, and the information sources differ by generation. These findings provide information for stakeholders interested in vaccine hesitancy, health informatics, messaging strategies, health literacy, and future health information outreach programs during epidemics or pandemics. Dissemination of public health information should include multiple information sources to reach all individual preferences across different generations.

## Introduction

### Background

#### Information Sources on COVID-19 Vaccination

The sources of information individuals consult and rely on have been linked to decisions on vaccination [1]. Nontraditional information sources such as social media and word of mouth vary in their reliability and verifiability and are not held to the same standards of peer review or fact-checking in the same way as information from government, news outlets, and health care [2]. Information and misinformation about COVID-19 can have dangerous consequences [3], because of the novelty of the disease and changes in information as more evidence is produced [4].

Studies in Sierra Leone and Italy have shown that the type and number of information sources used can significantly impact vaccine intention and uptake [5,6]. In an earlier study using the California Health Interview Survey (CHIS), people who reported using health care providers as their primary information source about vaccines have higher odds of human papillomavirus vaccine receipt or intention [7]. Another study found that obtaining vaccine information from the internet or relatives is associated with lower vaccination rates for measles, mumps, and rubella, and hepatitis B virus [1]. Additionally, when individuals used more than one information source to make measles, mumps, and rubella, or hepatitis B virus vaccine decisions, there was higher reported vaccine hesitancy [1]. On the contrary, a more recent study found that relying on and consulting more sources was associated with higher odds of COVID-19 vaccine uptake [8].

#### Vaccination Hesitancy and Uptake

Access to vaccinations and vaccination hesitancy (ie, the refusal or reluctance to receive vaccinations despite availability) are independent and joint contributors to vaccine uptake [9]. Vaccine hesitancy has been a persistent public health problem since the inception of vaccines [10], and in 2019, it was identified by the World Health Organization (WHO) as one of the top 10 global health threats [11]. Mass media, the technology used to reach the public such as radio, television, or newspapers, has historically played a significant role in influencing health beliefs and behaviors, including vaccine hesitancy and uptake [10,12].

#### Vaccination Uptake, Hesitancy, and Age

In 2021, vaccination uptake differed by generation, with coverage lowest among individuals aged 18 to 29 years and highest among individuals aged 65 years or older [13]. Prior literature indicates that younger adults tend to be more vaccine hesitant than members of older generations [10,12]. Few papers before the COVID-19 pandemic examined the role of information sources in vaccine uptake, perhaps because the issues were less politicized, people trusted information about vaccines from the health care and government sectors, and vaccine rates were higher overall [14]. With the COVID-19 pandemic, misinformation about vaccines became more prevalent and the question of vaccine uptake became polarized with political affiliation [14]. In one paper, social media use, often associated with younger age, is associated with higher odds of COVID-19 vaccine hesitancy [12]. There is also evidence that identified sources of information including government, science, and discussing vaccination with family and friends positively influenced COVID-19 vaccine intention [10]. However, to our knowledge, no studies have explicitly examined COVID-19 vaccine uptake by generation and information source.

Generational membership including Generation Z (Gen Z; born 1995‐2012), millennials (born 1980‐1994), Generation X (Gen X; born 1965‐1979), baby boomers (born 1946‐1964), and the Silent Generation (born 1925‐1945) is associated with varying historical, technological, social, economic, and political circumstances, which may impact the amount and type of information sources consumed [15,16]. Media preferences and trust vary by age group with one nationally representative survey study finding that 9.5% of Gen Z participants trust their social media contacts mostly to completely (Facebook and Twitter, subsequently rebranded X) for COVID-19 information, compared to 3.8% of Silent Generation, 6.3% of millennials, 5.9% of Gen X, and 6% of baby boomer participants [15]. In the same study, 44.6% of Gen Z participants agreed or strongly agreed with the statement “COVID-19 vaccines have many known harmful side effects,” compared to 44.7% of millennials, 43.2% of Gen X, 31.8% of baby boomers, and 18.7% of the Silent Generation participants [15]. However, no studies to our knowledge have examined the potential effect modification of the association between media preferences and vaccine uptake by age or generational status.

Given this context of varying trusted information sources and vaccine decision making, the WHO and Centers for Disease Control and Prevention (CDC) have monitored public communication on digital and nondigital platforms related to COVID-19 to address misinformation and promote accurate public health information [17,18]. These include social media, online platforms, news, radio, and television [17]. The WHO described an excess of both accurate and inaccurate information as an “infodemic” requiring coordinated management and has since created a framework to identify and respond to misinformation [17]. As a result, the CDC formed an Insights Unit to track and analyze information using a social listening protocol, resulting in reports that guided public health messaging [18].

### Gaps in the Literature

Taken together, previous literature includes investigations of the influence of different information sources on vaccination decisions across various demographics, yet there remain gaps in understanding these dynamics across the range of information sources at the population level. Studies are also needed to focus on the number of information sources as well as the type [19] and to consider differences in these associations by generation.

### Goal of This Study

To address these gaps, the current study leverages CHIS, a population-based California survey, to examine associations between the type and number of the most relied upon information sources and COVID-19 vaccination status. Most relied upon sources were a specific term created by CHIS referring to the highest ranked source. We hypothesized that the use of specific types of information sources such as “your doctor” is associated with higher rates of vaccination when compared to traditional news media (eg, television, radio, and newspaper). We also tested the effect modification of the relationship between information sources (type and number) and vaccination status by generational membership, hypothesizing that the impact of traditional news media sources on vaccination status will be greater among older adults and the impact of digital media sources (eg, social media) on vaccination status will be greater among younger adults. Additionally, we hypothesized that identifying social media as the most relied upon information source would be associated with lower odds of vaccination. The study findings can improve our understanding of media preferences as influences on health decision behaviors and vaccine hesitancy and inform public health messaging efforts tailored to generational differences.

## Methods

We conducted a secondary analysis of cross-sectional data from the 2022 CHIS.

### Data Source

The CHIS is a population-based survey of noninstitutionalized Californians collected by Social Science Research Solutions, a research company, for the University of California, Los Angeles Center for Health Policy Research [20]. The 2021‐2022 CHIS data were collected between March 18, 2021, and November 30, 2022, using a mixed-mode method surveying (phone and internet) of the representative population of Californians and geographically stratified address-based sampling. Of the adult interviews conducted, 89.5% were completed online, and 10.5% were over the telephone. CHIS strategically over-samples to account for households abstaining from participation and added a prepaid cell phone sample to reach individuals left out of general address-based sampling frameworks. Interviews were held in the following languages: Spanish, English, Chinese (Cantonese and Mandarin), Korean, Tagalog, and Vietnamese.

### Sample

The study sample included all adult Californians responding to the 2021‐2022 CHIS survey (N=21,463) [20]. No additional exclusion criteria were applied and there were no missing data.

### Ethical Considerations

The University of California Davis Institutional Review Board reviewed this study and determined it was not human participants research and therefore was exempt from further review based on the use of publicly available data without identifying information (IRBNet ID 2233309). As a secondary analysis of existing data, informed consent was not obtained by the study investigators. Information about informed consent of CHIS participants is available online [20].

### Variables and Measurement

#### Dependent Variable

Vaccine uptake, operationalized as self-reported vaccination status, is the dependent variable for both aims. Participants were asked, “Have you been fully vaccinated, partially vaccinated, or are you not vaccinated, for COVID-19?” Being fully vaccinated was defined in the CHIS as receiving 2 shots of the Pfizer or Moderna vaccine, 1 shot of the Johnson & Johnson vaccine, or 2 shots of the AstraZeneca or Sinovac vaccine (which are available in other countries, not in the United States). Partially vaccinated, not explicitly defined by the survey documentation, was interpreted to mean a vaccination series that had been initiated but not completed. Vaccinations were still being distributed to priority groups, through November 2022; therefore, to fully capture intent to vaccinate in our cohort, we categorized any level of vaccination (partial or full) as “Vaccinated” (yes=1) and “Not Vaccinated” as the reference group (no=0).

#### Independent Variables

Participants were asked about their most relied upon sources of COVID-19 information, categorized into 12 possible categories: newspapers, radio, television, family, friends, community leaders, religious leaders, social media, doctors, governmental agencies, places of employment, and none of the above. For the examination of the type of information source, the response options were recoded into 6 categories: traditional news media (television, radio, and newspapers), word of mouth (family, friends, religious leaders, and community leaders), social media, your doctor, your employer, or governmental agency, or none of these. The reference group was reporting traditional news media as the most reliable source. For the number of sources, the independent variable is a count of the original 12 information sources with a range of 0 to 11.

#### Covariates

Sociodemographic variables were included in the models as potential confounders. This included age categorized by generational membership, which was also examined as an effect modifier: Gen Z (reference group; aged 12‐25 y), millennials (aged 26‐39 y), Gen X (aged 40‐54 y), baby boomers (aged 55‐74 y), and Silent Generation (aged ≥75 y) [16]. The University of Southern California generational age ranges generally matched the CHIS age categories with some exceptions around cut-off years, for instance, the millennial category featuring young members from Gen X.

Following Joshi et al’s [21] conceptual framework on the factors that influence vaccine acceptance and hesitancy, the covariates were selected as relevant to this study and as important social and structural determinants of COVID-19 vaccination uptake [22]. They include race or ethnicity, urbanicity, self-reported sex, educational attainment, marital status, insurance status, and income. Reported race and ethnicities include White non-Hispanic (reference group), Hispanic, African American or non-Hispanic, American Indian or Alaskan Native, Asian, and Other or Two or More Races. Urbanicity was categorized as urban (reference group), mixed urbanicity, suburban, and rural. Self-reported sex was categorized as male (reference group) or female. Educational attainment was categorized as having no high school diploma (reference group), a high school diploma, some college degree, bachelor’s degree, master’s degree, or PhD. Marital status was categorized as married or living with a partner (reference group), widowed, divorced, separated, or never married. Insurance status was categorized as yes (reference group). Income was recoded into four categories: (1) less than US $39,999 (reference group); (2) US $40,000 to $79,999; (3) US $80,000 to $149,999; and (4) US $150,000 to over US $180,000.

### Statistical Analysis

All analyses were conducted with Stata 18 (StataCorp LLC) and CHIS survey weights were applied to account for the complex survey sampling design. We used descriptive statistics to summarize all study variables and to compare vaccination status by generation. Multivariable logistic regression was used to model information sources first as categories, then as counts, as functions of vaccination status in models adjusted for all covariates. We also tested the effect modification of these associations by generational membership by adding information source by generation interaction terms to the models. If any of the source by generation interaction terms were significant, we conducted and reported stratified models by generation. Statistical significance was set at *P*<.05.

## Results

### Sample Description

Before applying survey weights, most participants were baby boomers aged 55 to 74 years (n=8905, 41.49%), followed by Gen X aged 40 to 54 years (n=5328, 24.82%), and millennials aged 26 to 39 years (n=3598, 16.76%; Table 1). Most participants were female (n=12,238, 57.02%). Over half held a master’s degree or PhD (54.5%, n=11,699) and 33.2% (n=7116) had some college, vocational education, or an associate’s degree. Almost half of the participants identified as White, non-Hispanic (n=10,432, 48.6%), followed by Hispanic (n=5719, 26.7%), Asian (n=3234, 15.1%), African American (n=1072, 5%), American Indian or Alaska Native (n=129, 0.6%), and two or more races (n=877, 4.1%). Less than half lived in urban areas (n=8956, 41.7%) and 31.9% (n=6855) lived in suburban areas. More than half of the participants were married (n=12,104, 56.4%) and 33.1% (n=7107) had an annual income greater than or equal to US $150,000. Most participants had health insurance (n=20,666, 96.3%). Just over half relied on traditional news media (ie, television, radio, and newspaper) as their primary source of COVID-19 information (n=11,356, 52.9%).

**Table 1.** Sociodemographic characteristics and COVID-19 information sources among Californian adults, 2021‐2022 (N=21,463).

Characteristics	Value, n (%)
Age by generation (y)	
Generation Z (18-25)	1106 (5.15)
Millennials (26-39)	3598 (16.76)
Generation X (40-54)	5328 (24.82)
Baby boomers (55-74)	8905 (41.49)
Silent Generation (≥75)	2526 (11.77)
Sex	
Male	9225 (42.98)
Female	12,238 (57.02)
Education	
No high school diploma	558 (2.60)
High school diploma	553 (2.58)
Some college (vocational or AS or AA included)	7116 (33.15)
BA or BS degree	1537 (7.16)
MA or MS or PhD	11,699 (54.51)
Race or ethnicity	
African-American, non-Hispanic	1072 (4.99)
American Indian or Alaska Native, non-Hispanic	129 (0.6)
Asian, non-Hispanic	3234 (15.07)
Hispanic	5719 (26.65)
White, non-Hispanic	10,432 (48.60)
Two or more races	877 (4.09)
Urbanicity or rurality	
Urban	8956 (41.73)
Mixed	2038 (9.50)
Suburban	6855 (31.94)
Rural	3614 (16.84)
Marital status	
Never married	4288 (19.98)
Married or living with partner	12,104 (56.39)
Widowed or separated or divorced	5071 (23.63)
Household income (annual, US $)	
<39,999	5556 (25.89)
40,000‐79,999	5055 (23.55)
80,000‐149,999	3745 (17.45)
>150,000	7107 (33.11)
Has health insurance	20,666 (96.29)
Most relied upon source of COVID-19 information	
Traditional news media (television, radio, and newspaper)	11,356 (52.91)
Word of mouth (family, friends, community, and religious leaders)	1171 (5.46)
Social media	2959 (13.79)
Your doctor	1013 (4.72)
Governmental agency or employer	3733 (17.39)
None of these	1231 (5.74)

Most participants were vaccinated for COVID-19 (n=19,836, 92.4%), and the vaccination rates increased slightly with age (from 88.97% among Gen Z to 96% in the Silent Generation). Among survey respondents who were vaccinated, the most relied upon information sources were their employer or government (95%) and traditional news media (94.6%). Vaccination rate was lowest (n=936, 76%) among those reporting no sources of information, whereas the highest vaccination rates (93%‐95%) were found for those reporting between 2 and 7 resources (Table 2). Among survey respondents who did not use any information sources, 76% (n=936) were vaccinated for COVID-19, whereas this rate was 80.6% (n=29) among those who used 11 resources.

**Table 2.** COVID-19 vaccination status by source count (N=21,463).

Source count	Total (N=21,463)	Vaccinated (n=19,836), n (%)
0	1231	936 (76.04)
1	5085	4665 (91.74)
2	4630	4334 (93.61)
3	4096	3890 (94.97)
4	2726	2562 (93.98)
5	1720	1618 (94.07)
6	941	880 (93.52)
7	554	517 (93.32)
8	266	243 (91.35)
9	132	122 (92.42)
10	46	41 (89.13)
11	36	29 (80.56)

### Regression of Vaccination Status on Information Source Type

In the fully adjusted logistic regression model of information source type and vaccination status (Table 3), compared to respondents using traditional news media for information about COVID-19, those relying on word of mouth had 40% lower odds (*P*<.002); those using social media had 38% lower odds (*P*<.001); those relying on their doctors had 59% lower odds (*P*<.001); and those reporting “none of these” had 79% lower odds of being vaccinated for COVID-19 (*P*<.001). There were no significant differences between using employers or governmental agencies and receiving a COVID-19 vaccination compared to traditional news media.

**Table 3.** Multivariable logistic regression models of vaccination status^a^.

	Information sources and vaccine status			Count of information sources and vaccine status		
	OR^b^	*P *value	95% CI	OR	*P *value	95% CI
Source count	—^c^	—	—	1.09	<.001	1.04‐1.15
Relied upon information sources						
Traditional news media	—	—	—	—	—	—
Word of mouth	0.60	.002	0.44‐0.82	—	—	—
Social media	0.62	<.001	0.51‐0.77	—	—	—
Your doctor	0.41	<.001	0.29‐0.57	—	—	—
Governmental agency or employer	1.05	.72	0.79‐1.39	—	—	—
None of these	0.21	<.001	0.16‐0.28	—	—	—
Age by generation (y)						
Generation Z (18-25)	—	—	—	—	—	—
Millennials (26-39)	1.01	.95	0.75‐1.35	1.06	.70	0.78‐1.44
Generation X (40-54)	1.34	.09	0.95‐1.88	1.45	.04	1.03‐2.05
Baby boomers (55-74)	2.72	<.001	1.93‐3.83	3.20	<.001	2.27‐4.50
Silent Generation (≥75)	3.62	<.001	2.30‐5.71	4.35	<.001	2.76‐6.86
Annual household income (US $)						
<39,999	—	—	—	—	—	—
40,000‐79,000	1.36	.009	1.08‐1.72	1.39	.006	1.10‐1.75
80,000‐149,000	1.69	<.001	1.29‐2.20	1.67	<.001	1.29‐2.18
150,000->180,000	2.37	<.001	1.90‐2.95	2.36	<.001	1.91‐2.92
Has health insurance	0.75	.08	0.55‐1.04	0.75	.07	0.56‐1.02
Race or ethnicity						
African-American, NH^d^	0.60	.004	0.43‐0.85	0.60	.002	0.43‐0.83
American Indian or Alaska Native, NH	0.76	.57	0.30‐1.93	0.68	.41	0.28‐1.70
Asian, NH	4.25	<.001	2.84‐6.36	4.27	<.001	2.85‐6.40
Hispanic	1.28	.008	1.07‐1.54	1.31	.004	1.09‐1.56
White, NH	—	—	—	—	—	—
Two or more races	1.66	.004	1.18‐2.35	1.67	.005	1.18‐2.38
Marital status						
Married or living with partner	—	—	—	—	—	—
Widowed or separated or divorced	0.98	.84	0.80‐1.20	0.99	.94	0.80‐1.22
Never married	1.35	.02	1.04‐1.76	1.31	.04	1.01‐1.69
Urbanicity						
Urban	—	—	—	—	—	—
Mixed	0.56	<.001	0.42‐0.75	0.57	<.001	0.43‐0.76
Suburban	0.84	.06	0.69‐1.01	0.83	.049	0.69‐0.99
Rural	0.45	<.001	0.36‐0.57	0.44	<.001	0.35‐0.55
Self-reported sex	1.14	.10	0.98‐1.32	1.19	.04	1.01‐1.39
Educational level						
No HS^e^ diploma	—	—	—	—	—	—
HS diploma	0.46	.003	0.29‐0.76	0.47	.003	0.29‐0.77
Some college (vocational or AS or AA included)	0.66	.07	0.43‐1.03	0.64	.045	0.41‐0.99
BA or BS degree	0.82	.48	0.48‐1.42	0.79	.38	0.47‐1.33
MA or MS or PhD	1.70	.04	1.03‐2.78	1.66	.045	1.01‐2.72

a All estimates are survey weighted and generalized to a sample of N=29,560,694.

b OR: odds ratio.

c Not applicable.

d NH: non-Hispanic.

e HS: high school.

### Regression of Vaccination Status on Information Source Count

In the fully adjusted model examining the count of relied upon information sources and vaccination status, each additional source was associated with 9% higher odds of being vaccinated for COVID-19 (*P*<.001; Table 3). In this model, the odds of being vaccinated were higher among Gen X, baby boomers, and Silent Generation when compared to Gen Z. Covariates in this model associated with higher odds of vaccination included an annual income of over US $40,000 (compared to <US $40,000), self-reporting race or ethnicity as Hispanic, Asian, or two or more races (compared to White non-Hispanic), never being married (compared to married or living with a partner), self-reporting male, and attaining postgraduate education (compared to no high school diploma). Covariates associated with lower odds of vaccination in the source count model included self-reporting race or ethnicity as African American (compared to White non-Hispanic), living in a rural or suburban environment (compared to urban), and attaining a high school diploma or completing some college (compared to no high school diploma).

### Effect Modification

We found significant effect modification of the associations of type of information source and vaccination status by generation. Accordingly, we conducted full models stratified by generation (Table 4). For each generation, 2 models were run, one with information source type and one with information source count as the primary independent variable.

**Table 4.** Multivariable regression models of vaccination status stratified by generation^a^.

Information sources	Generation Z (18-25 y; n=1106)		Millennial (26-39 y; n=3598)		Generation X (40-54 y; n=5328)		Baby boomers (55-74 y; n=8905)		Silent Generation (≥75 y; n=2526)	
	Odds ratio (95% CI)	*P* value	Odds ratio (95% CI)	*P* value	Odds ratio (95% CI)	*P* value	Odds ratio (95% CI)	*P* value	Odds ratio (95% CI)	*P* value
Traditional news media	—^b^	—	—	—	—	—	—	—	—	—
Word of mouth	0.76 (0.41‐1.42)	.39	0.82 (0.36‐1.86)	.64	0.41 (0.23‐0.74)	.004	0.77 (0.44‐1.36)	.36	1.92 (0.26‐14.42)	.52
Social media	1.44 (0.84‐2.46)	.18	0.69 (0.45‐1.06)	.09	0.63 (0.40‐0.98)	.04	0.18 (0.11‐0.30)	<.001	0.16 (0.05‐0.51)	.003
Your doctor	0.30 (0.07‐1.30)	.11	1.00 (0.38‐2.63)	.99	0.29 (0.17‐0.50)	<.001	0.47 (0.33‐0.67)	<.001	0.56 (0.10‐2.97)	.49
Your employer or government agency	2.72 (0.93‐7.96)	.07	1.02 (0.62‐1.68)	.94	1.07 (0.65‐1.73)	.80	0.90 (0.60‐1.36)	.61	1.34 (0.58‐3.10)	.49
None of these	0.39 (0.18‐0.84)	.02	0.36 (0.16‐0.74)	.005	0.24 (0.13‐0.46)	<.001	0.09 (0.06‐0.13)	<.001	0.10 (0.04‐0.24)	<.001
Source count	1.18 (1.003‐1.38)	.045	1.02 (0.92‐1.13)	0.65	1.13 (1.03‐1.24)	.01	1.11 (1.001‐1.24)	.048	1.28 (0.99‐1.63)	.051

a All estimates are survey weighted and generalized to a sample of N=29,560,694.

b Not applicable.

In the sample restricted to Gen Z, the only information source type associated with vaccination status was “none of these,” which was associated with lower odds of being vaccinated compared to traditional news media (odds ratio [OR] 0.39, 95% CI 0.18‐0.84; Table 4). Source count was significantly associated with higher odds of vaccination uptake (OR 1.18, 95% CI 1.003‐1.38; Table 4). In the sample restricted to millennials, only “none of these” information sources (compared to traditional news media) was associated with lower odds of vaccination (OR 0.36, 95% CI 0.16‐0.74). Source count was not significantly associated with vaccination uptake for millennials. In the sample restricted to Gen X, word of mouth (OR 0.41, 95% CI 0.23‐0.74)*,* social media (OR 0.63, 95% CI 0.40‐0.98)*,* your doctor (OR 0.29, 95% CI 0.17‐0.50)*,* and “none of these” (OR 0.24, 95% CI 0.13‐0.46) were significantly associated with lower odds of vaccination compared to traditional news media. Source count was significantly associated with higher odds of vaccination for Gen X (OR 1.13, 95% CI 1.03‐1.24).

In the sample restricted to baby boomers, social media (OR 0.18, 95% CI 0.11‐0.30), your doctor (OR 0.47, 95% CI 0.33‐0.67), and “none of these” (OR 0.09, 95% CI 0.06‐0.13) were significantly associated with lower odds of vaccination compared to traditional news media. Source count was significantly associated with higher odds of vaccination for baby boomers (OR 1.11, 95% CI 1.001‐1.24). In the sample restricted to the Silent Generation, social media (OR 0.16, 95% CI 0.05‐0.51) and “none of these” (OR 0.10, 95% CI 0.04‐0.24) were associated with lower odds of vaccination when compared to traditional news media. Source count approached but did not reach statistical significance for the Silent Generation.

## Discussion

### Principal Findings

In this large population-based sample of adult Californians, we find that both the identified most-relied-upon information source and the count of identified sources are robust predictors of the COVID-19 vaccination status. This supports previous evidence that information sources are associated with vaccination status [1,5]. This study contributes new knowledge to the associations between vaccine status, number of sources used, source type, and examines variation in these associations by generational membership. Thus, these are important factors to consider in the future during research and public health program development when the need arises.

In line with previous literature that found social media to be associated with negative views towards COVID-19 vaccination receipt, we found that use of social media was associated with lower odds of vaccination status compared to traditional news media and controlling for covariates. When stratified by generation, this association held among Gen X, baby boomers, and the Silent Generation. This divergence may be explained by the personalization of social media feeds and the potential for algorithmic information silos. Similar to our findings, Moon et al [12] found higher social media use was a significant predictor of COVID-19 vaccine hesitancy. In this study, this was true across older generations such as Gen X, baby boomers, and the Silent Generation. Future research may be warranted to explore how targeted advertising or recommended pages via an algorithm may be able to amplify vaccination information to combat the spread of misinformation.

In this analysis, we relied upon traditional news media as the reference category. We found in all generational groups, with the exception of Gen Z and millennials, that relying on social media for vaccine information compared to traditional news media was associated with lower odds of vaccination uptake. Among Gen Z and millennials, relying on social media compared to traditional news media was not associated with vaccination uptake. This means that although these generational groups tend to use social media more than others, its influence is not different than that of traditional news media. These groups may view traditional news media through social media platforms, thereby minimizing the difference in the influence of these information sources. While other researchers have identified relationships between social media use and vaccine hesitancy [12], they were unable to detail what type of content was being consumed through social media. Social media may include short-form clips of traditional news media information. These inconsistencies warrant further research to better understand how different generations consume social media and how this, in turn, may impact vaccination uptake.

Our findings consistently showed that respondents reporting “none of these” information sources had lower odds of being vaccinated across all generations. The deeper meaning behind this response is unclear, though it could represent those who abstain from media or do not interact with the listed sources; this may represent a gap in the listed responses to the survey question. For instance, alternate popular sources such as podcasts were not among the multiple-choice answers provided by CHIS. It is also possible that those who did not fully understand the question or preferred not to answer selected this option.

While reporting “your doctor” as the most relied upon information source was associated with lower odds of vaccination, this was in comparison to traditional news media. At the time of this survey, information surrounding COVID-19 and vaccinations was communicated heavily through various traditional and nontraditional media sources. This finding does not indicate that doctors were not perceived as reliable sources of information for COVID-19, but rather that traditional news media sources likely played a more central role in distributing COVID-19 vaccination information and related public health messaging.

Consistent with past studies, we found that relying on a higher number of information sources was associated with higher COVID-19 vaccination rates, possibly due to accumulating greater knowledge about vaccine-related information [7,8]. Our results further identified a dose-response relationship with a greater quantity of relied-upon information sources being associated with increasing odds of being vaccinated for COVID-19. These results suggest that people who engage in more health information-seeking behaviors may be more successful in garnering accurate and updated public health advice. It is also plausible that individuals seeking multiple sources of information have a greater propensity to be vaccinated due to other personal characteristics allied with health promotion.

It should be mentioned that the vaccination rates across each generation in this sample surpassed both state and national averages [23]. This study found that Gen Z had the lowest COVID-19 vaccination rates among all generations, while the Silent Generation had the highest. In comparison, California data shows that individuals aged 40 to 69 years exhibited both higher hesitancy and higher likelihood of vaccination compared to other age groups [12]. The concept of generational imprinting can suggest that the generation that grew up during poliovirus vaccination development may view COVID-19 vaccinations in a positive light and therefore may desire to receive the vaccination as soon as they can [15]. The high rate of Silent Generation vaccine receipt in our sample may be explained by the timing of the survey, during which vaccine rollout was made available to older adults and health care workers first.

These findings on COVID-19 vaccination patterns among Californians have important public health implications about the role of information sources. They illuminate the need to tailor messaging by generation based on preferred information sources. This will support accurate and trustworthy information reaching all audiences, as the sources of information used are linked to knowledge, vaccine intention, and vaccination rates. Accessing a variety of sources may contribute to shaping knowledge and behaviors regarding vaccination. Reliance on diverse information sources could combat algorithmic information silos, with those who access multiple, reliable sources being more likely to be informed about the benefits and risks of vaccines and thereby influencing their vaccination decisions. This information contributes to our understanding of the information sources used by age generations in the context of the COVID-19 pandemic and may inform strategies for promoting health equity and widespread immunization in similar, future health contexts.

### Limitations

This study had several limitations. First, due to the cross-sectional study design, we cannot establish temporal precedence or causation. Second, the data were collected during the COVID-19 pandemic, and we cannot conclude that the findings would hold before or after the pandemic. Third, because the CHIS data were collected in 2021 and 2022, there was not equal access to vaccination for all participants as vaccines were just becoming available to the public. Accordingly, we combined those who were partially vaccinated with those who were fully vaccinated into one “vaccinated” category. Partial vaccination does not necessarily indicate an intention to become fully vaccinated. We have no assurance that partially vaccinated individuals completed the vaccination series, and if they did not, we do not know whether vaccine hesitancy was a contributing factor. Additionally, COVID-19 vaccination status is time-dependent, as vaccine availability differed across age groups and months in 2021 and 2022 when the data were collected. The CHIS dataset does not include the interview month, resulting in the inability to adjust for survey timing. Combining these categories may introduce misclassification bias and may inflate the rates of vaccination in the study and underestimate underlying vaccine hesitancy. Also, we are unable to identify the amount, quality, or precise source of information in any of the relied upon categories. For instance, social media platforms include a variety of information sources such as clinician influencers, friends and family, public health, and traditional news.

Fourth, we can only speculate about the information source choice “none of these” and what this meant to respondents. Our finding that this choice was consistently associated with lower odds of vaccination warrants follow-up in future studies, perhaps with cognitive interviews or other qualitative approaches designed to more fully understand this selection. This information would be helpful in informing public health guidance and messaging, building trust within community-academic partnerships, and efforts to improve health literacy in the community.

Fifth, because this is a secondary data analysis, our findings are subject to unmeasured confounding. Greater information about vaccination intentions could have further elucidated the findings given that these data were collected in the early phases of vaccine rollout. In addition, the CHIS categorical age ranges fit the generational age cutoffs closely with a few exceptions [16]. For instance, millennials are defined as those born between 1980 and 1994, corresponding to those aged 28 to 42 years in 2022. However, the CHIS survey had categorical age ranges that did not perfectly fit (26-29 y, 30-34 y, and 35-39 y) these generational groupings. As a result, our generational membership included or missed some members with ages close to the cut points, for example, millennials included the young members of Gen X. In addition, there may be limitations to generalizability as a result of the “healthy user effect” or positive selection bias [24]. Our sample had higher than average vaccination rates, which may relate to participants being more likely to participate in a study about health [23,24]. As a result, our sample may have different characteristics from the population of interest, as the vaccination rates in our sample were higher than the California state average [23]. Finally, because the CHIS survey is a population-based sample of California residents, findings may not be generalizable beyond California.

Today’s political climate has seen a rise in antivaccine attitudes [25] alongside the perpetuation of false narratives linking vaccines to autism in children [26]. These events may risk delaying the development of new vaccines and undermining public confidence in vaccine experts. Within this context, a deeper understanding of how information sources influence vaccine decision-making may be more pertinent than ever.

### Conclusions

Our findings suggest that an effective approach to disseminating accurate public health information should include multiple methods to reach all generational preferences of the public. The findings from this study on COVID-19 vaccination patterns in California provide important contributions for stakeholders interested in vaccine hesitancy, health informatics, and health literacy. They identify the importance of understanding generational differences in how people rely on media during a pandemic. These findings may also not be limited to vaccination use, and further research could expand to explore other preventative health care behaviors such as screenings, or other public health aspects that are discussed by media sources.

## Abbreviations

CDC: Centers for Disease Control and Prevention
CHIS: California Health Interview Survey
OR: odds ratio
WHO: World Health Organization
